# Pre-Clinical Assessment of Roflumilast Therapy in a Thoracic Model of Spinal Cord Injury

**DOI:** 10.3390/pharmaceutics15051556

**Published:** 2023-05-21

**Authors:** Carla S. Sousa, Rui Lima, Jorge R. Cibrão, Eduardo D. Gomes, Luís S. Fernandes, Tiffany S. Pinho, Deolinda Silva, Jonas Campos, António J. Salgado, Nuno A. Silva

**Affiliations:** 1 Life and Health Sciences Research Institute (ICVS), School of Medicine, University of Minho, 4710-057 Braga, Portugal; 2 ICVS/3B’s Associate Lab, PT Government Associated Lab, 4805-017 Guimarães, Portugal; 3 Department of Neurosurgery, Hospital Garcia de Orta, 2805-267 Almada, Portugal

**Keywords:** Roflumilast, spinal cord injury, motor recovery, rat model

## Abstract

The failure of axons to regenerate after a spinal cord injury (SCI) remains one of the greatest challenges in neuroscience. The initial mechanical trauma is followed by a secondary injury cascade, creating a hostile microenvironment, which not only is not permissive to regeneration but also leads to further damage. One of the most promising approaches for promoting axonal regeneration is to maintain the levels of cyclic adenosine monophosphate (cAMP), specifically by a phosphodiesterase-4 (PDE4) inhibitor expressed in neural tissues. Therefore, in our study, we evaluated the therapeutic effect of an FDA-approved PDE4 inhibitor, Roflumilast (Rof), in a thoracic contusion rat model. Results indicate that the treatment was effective in promoting functional recovery. Rof-treated animals showed improvements in both gross and fine motor function. Eight weeks post-injury, the animals significantly recovered by achieving occasional weight-supported plantar steps. Histological assessment revealed a significant decrease in cavity size, less reactive microglia, as well as higher axonal regeneration in treated animals. Molecular analysis revealed that IL-10 and IL-13 levels, as well as VEGF, were increased in the serum of Rof-treated animals. Overall, Roflumilast promotes functional recovery and supports neuroregeneration in a severe thoracic contusion injury model and may be important in SCI treatment.

## 1. Introduction

Spinal cord injury (SCI) has a dramatic impact on motor, sensory and autonomic functions [1]. Currently, SCI is managed only through surgery decompression and maintaining the mean arterial blood pressure [2,3]. The use of steroids as a standard of care is currently highly controversial [4]. As a result, it is crucial to develop novel therapeutic strategies to repair spinal cord injuries.

SCI is characterized by a complex pathophysiology. Initial mechanical trauma is followed by damaging secondary injury events that lead to further injury, creating an adverse microenvironment for regeneration [5,6]. A major discovery in this field has been the identification of inhibitory proteins in CNS myelin. In 1988, Schwab and colleagues provided the first direct evidence that CNS myelin contains proteins that inhibit neurite outgrowth in vitro [7]. Nogo-A, myelin-associated glycoprotein (MAG) and oligodendrocyte myelin glycoprotein (OMgp) are among these proteins. They have no sequence similarity or structural homology, yet surprisingly they all bind to a common receptor complex to mediate inhibition. These inhibitors are responsible for the Rho-mediated activation of downstream effectors, such as Rho-associated kinase (ROCK), which then induces actin polymerization, leading to growth cone collapse and the inhibition of neurite outgrowth [8,9]. MAG, Nogo and OMgp signal through a common pathway, so inhibiting protein kinase C PKC, Rho and ROCK is logical to promote axonal regeneration. However, it is also possible to manipulate neurons at the molecular level so that they no longer respond to myelin inhibitors. The approach is based on the observation that MAG initially permits neurite outgrowth and only becomes inhibitory beyond a certain developmental time point. In 2001, Cai et al. demonstrated a direct correlation between neuronal adenosine monophosphate (cAMP) levels and the inhibition of neurite outgrowth by MAG and myelin. The levels of endogenous cAMP are high in P1 dorsal root ganglion (DRG) neurons, and substantial neurite outgrowth is observed despite the presence of MAG and myelin; however, at P3−4, there is an abrupt decrease in neuronal cAMP, which coincides with myelin inhibition [10]. These findings raise the possibility that pharmacological agents could be used to elevate cAMP levels in neurons, thereby increasing their ability to regenerate.

Many promising treatments have resulted from the identification of cAMP as a modulator of axonal regeneration [11], some of which use phosphodiesterase-4 (PDE4) inhibitors [11]. However, of all the mediators that have been tested to date, possibly the most significant finding was that acute administration of Rolipram alone significantly improved axonal integrity and functional outcome post-SCI [12,13,14]. Unfortunately, Rolipram has been abandoned for human use because of its side effects [10,15]. However, this does not undermine the therapeutic potential of second-generation PDE4 inhibitors. For instance, it was demonstrated that Roflumilast (Rof) has the same binding site as Rolipram and can increase the intracellular levels of cAMP. Additionally, Rof presents excellent pharmacokinetic properties, such as rapid absorption and distribution, linear kinetics, good bioavailability and low toxicity [16], and has already been approved for human use in chronic obstructive pulmonary disease (COPD) [17]. Moreover, it was demonstrated that Rof was able to protect the neural tissue after cerebral ischemia in rats by modulating the immune response. Roflumilast administered intraperitoneally decreases the expression of pro-inflammatory cytokines and protects the neurons in the brain [18]. Additionally, Rof is also able to change the microglia phenotype from a pro-inflammatory status to a pro-regenerative one, both in vitro and in vivo [19]. Taking into account all the above-mentioned properties, herein we aimed to study the therapeutic action of Roflumilast in a severe thoracic SCI rat model.

## 2. Materials and Methods

### 2.1. Spinal Cord Cultures

To evaluate if Roflumilast was able to induce specific cAMP-signaling markers, we employed an in vitro culture system composed of mixed cells of spinal cord isolated from Wistar Han pups within 5 days (P5). Spinal cords were isolated by hydraulic extrusion according to [20]. Cells were kept for 7 days in Neurobasal^TM^ medium with 2% B27, 1% Pen/Strep, 1% L-Glutamine and Glial-derived neurotrophic factor (GDNF) at 10 ng/mL. Cells were then exposed for 48 h to Roflumilast at 20 uM in Neurobasal^TM^ medium with 1% Pen/Strep. Controls had exposure to the same media without Roflumilast. Cells were fixed in cold 4% PFA for 10 min, then triple-immunolabeled for b3 tubulin, cAMP protein kinase (cAMPKA) and the cAMP response element binding protein (CREB). Four wells for each group were automatically imaged, focusing on neuronal enriched regions of interest (ROIs). Acquisition settings such as exposure time and detector gain were kept constant throughout groups. A total of 40 photomicrographs per condition(10/well) at 20× magnification were taken on an inverted widefield microscrope (Olympus IX81, Tokyo, Japan). Image analysis was performed in FIJI with an automatic macro that used the b3 tubulin-labeled neuronal cell bodies to segment the cytoplasmic cAMPKA and the nuclear CREB. Measurements of the area of each marker of interest were taken and normalized to the number of positive b3 tubulin-labeled cells.

### 2.2. Organotypic Cultures

To assess the effect of Roflumilast on axonal regeneration, we used organotypic cultures of spinal cord slices (350 µm) from the thoracic region of Wistar Han pups within 6 days (P6). The spinal cords were dissected under aseptic conditions and placed on cold HBSS containing 1% Pen/Strep. Then, 350 µm slices were obtained using a tissue chopper. Preserved spinal cord slices were selected under magnification. The slices were then divided into two groups with or without 20 nM of Rof in Neurobasal^TM^ medium with 2% B27, 1% Pen/Strep, 1% L-Glutamine and 6 mg/mL of glucose (medium was changed every 2 days for both groups). Finally, slices were fixed and immunolabeled for neurofilament following the protocol described below. The area occupied by the axons in each spinal cord slice explant was calculated using the ImageJ (NIH) plugin Neurite-J. Our analysis was focused only on the neurofilament that was detected outside the spinal cord slice, measuring the newly growing axons instead of the total area of neurofilament because there were some minor differences in the area of the spinal cord slice alone. Briefly, the image was automatically translated to eight bits, and a binary mask was created with the aid of the “Analysis Particles” function, which enables the correct segmentation of axonal structures based on an intensity-threshold image coupled with morphological parameters such as structure size and circularity. The mask generated can then be added as input to the Neurite-J plugin.

### 2.3. ELISA Assay

For the protein extraction, samples were prepared in RIPA buffer (Tris-HCl 20 mM; NaCl 150 mM; EDTA 5 mM; 0.1% SDS). All samples were tested in duplicate. We used an ELISA kit for cAMP (Enzo Complete cAMP ELISA Kit). After the wells were filled with the respective samples, adequate controls and anti-cAMP antibodies, the plate was sealed and incubated for 2 h at room temperature with orbital agitation (500 rpm). Following incubation, the plate was washed, and cAMP conjugate and pNpp substrate were added to the wells to initiate the colorimetric reaction, which took 1 h at room temperature. The reaction was finished with the addition of ELISA Stop solution (HRP), and the optical densities were read at 405 nm using a spectrophotometer. cAMP concentration was inferred based on a four-parameter logistic calibration curve.

### 2.4. In Vivo Study Design

The objective of this study was to evaluate the therapeutic potential of Rof treatment after SCI. We assessed the effects of Rof treatment in a rat model, evaluating functional recovery and histological and molecular modifications. The number of animals in this study was determined using G*power software (12 animals per group). Fifteen animals per group were randomly treated with Roflumilast or saline, and all data were collected blind to the treatment group. All procedures were carried out in accordance with EU directive 2010/63/EU and were approved by the ethical committee in life and health sciences (Ref: DGAV 022405, SECVS116/2016, University of Minho, Braga, Portugal).

### 2.5. Spinal Cord Injury Model and Treatment

A total of forty-two Wistar Han female rats (8–10 weeks old, weighing 210–260 g) were used in this study (30 rats for the pre-clinical trial and 12 for cAMP quantification). Animals were kept under standard laboratory conditions (12 h light/12 h dark cycles, 22 °C, relative humidity of 55%, ad libitum access to standard food and water) and housed in pairs. Animal handling was performed 3 days prior to surgery.

The T8 contusion model was induced, as previously described [21,22]. General anesthesia was induced by an intraperitoneal (i.p.) injection of a ketamine (100 mg/mL, Imalgene/Merial) and medetomidine hydrochloride (1 mg/mL, Dormitor/Pfizer) mixture at a volume ratio of 1.5:1. Once anesthetized, animals received subcutaneous injections of the analgesic butorphanol (10 mg/mL, Butomidor/Richter Pharma AG) and the antibiotic enrofloxacin (5 mg/mL, Baytril/Bayer). The fur was shaved from the surgical site and the skin was disinfected with ethanol 70% and chlorohexidine. Surgical procedures were performed under sterile conditions. The animals were placed in a prone position and a dorsal midline incision was made at the level of the thoracic spine (T5–T12). The paravertebral muscles were retracted, and the spinous process of T5 was identified by observing a prominent vessel that was placed between the fourth and the fifth thoracic processes. Using the back edge of the scalpel blade, the spinous processes were counted from T5 until T8, and the latter was marked with a small cut. Then, the spinous process and laminar arc of T8 was removed, and the spinal cord was exposed. The dura was left intact. A weight drop trauma model was used, which consisted of dropping a 10 g weight rod from a 10 cm height onto the exposed spinal cord. The rod was guided through a stabilized tube positioned perpendicular to the center of the spinal cord. After the trauma, the muscles were sutured with Vicryl sutures (Johnson and Johnson^®^, New Brunswick, NJ, USA), and the incision closed with surgical staples (Fine Science Tools^®^, North Vancouver, BC, Canada). Anesthesia was inverted using atipamezole (5 mg/mL, Antisedan/Pfizer^®^, New York, NY, USA). One-hour post-injury, the animals were randomly divided into two experimental groups, the Roflumilast treatment group and the control that only received vehicle (saline). The post-operative care for all rats included butorphanol (Richter Pharma AG^®^, Wels, Austria) administration twice daily for a five-day period, and vitamins (Duphalyte, Pfizer^®^), saline and enrofloxacin (Bayer^®^, Leverkusen, Germany) twice daily for seven days. The bladders were manually expressed twice a day until animals recovered spontaneous voiding. A weekly body weight measurement was taken as a measure of general health. If a weight loss over 10% of body weight was detected, a high-calorie oral supplement (Nutri-Cal^®^, Bondurant, IA, USA) was administered daily.

**Figure 1:**
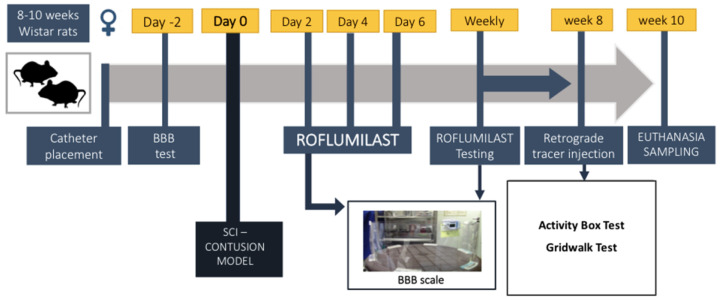
Methodological scheme and temporal line for the in vivo study design.

For Rof or saline administration, we inserted an intrathecal 28 G catheter 7 days before inflicting the contusion. Under general anesthesia, a dorsal midline incision was made. The spinous process and laminar arc of T13 were removed, the dura was gently opened and the catheter was introduced up to the level of T8 and then retracted to T9 (1 level below the level of contusion). The laminectomy previously performed at T8 exposed the spinal cord and was used as a window of observation to confirm that the tip of the catheter was rightly placed. At the skin, in the exit point, the catheter was fixed subcutaneously in one point, and the skin was closed with Vicryl sutures (Johnson and Johnson^®^), leaving approximately 1 cm of catheter length outside. In order to prevent the rats from reaching the catheters, the exit point was placed near the back of their heads. For repeated administrations, the catheter tip was heated to close its extremity each time after an injection. Before a new treatment administration, the catheter was opened by cutting approximately 1 mm off its extremity. Animals were functionally evaluated (Basso, Beattie and Bresnahan (BBB) scale) on day 2 post-procedure and were excluded if any motor disability was detected. Rof and saline were administered locally through the intrathecal catheter (100 µg/kg) on days 2, 4 and 6 post-injury and then weekly by intraperitoneal injection (1 mg/kg) until the eighth week. The selected dosage of Roflumilast was taken into account based on previously published work, namely, it was demonstrated that 1 mg/kg of Roflumilast administrated intraperitoneally had superior neuroprotective effects than 0.5 mg/kg in the cerebral ischemia injury model [18]. The methodological scheme and temporal line for the in vivo study can be found on Figure 1.

### 2.6. Behavioral Assessment

The Basso, Beattie and Bresnahan (BBB) locomotor rating scale was used to evaluate functional recovery, as previously described [23]. All behavioral tests were performed blind on the treatment groups. The BBB test was executed three days post-injury and weekly thereafter for a 7-week period. A BBB score of 0 indicates no movement of the hindlimbs. From 1 to 8, a BBB score indicates joint movement but no weight support. A BBB score from 9 to 20 indicates the ability to support weight and use the limb for locomotion but with some degree of abnormality. A BBB score of 21 corresponds to locomotion in a normal rat.

Activity Box Test (ABT)

Activity box tests measure distance traveled, velocity and number of rearing movements of animals to assess exploratory behavior. The test was conducted in an open arena (43.2 × 43.2 cm) with transparent acrylic walls (MedAssociates Inc., Fairfax, VT, USA). Animals started the test at the center of the arena and were allowed 5 min to explore it. Data were collected using activity monitoring software. Velocity was used as a measure of locomotor activity [24,25].

Gridwalk test (GWT)

The GWT assesses deficits in descending motor control by examining the ability of the rat to navigate across a 1.3 m long wire mesh walkway [26]. A training session was performed before surgery, in which groups of four animals freely explored the grid for 10 min. The test consisted of recording each rat’s performance in a 3 min trial, where the animals freely explored the grid. For each 3 min trial, we selected 30 s based on continuous movements of the animal on the grid and then determined the number of errors committed by the rat. The Gridwalk test was performed 8 weeks post-injury.

### 2.7. Fluoro-Gold–Retrograde Axonal Tracer

Retrograde tracing explores neuronal retrograde axonal transport and is commonly used to elucidate the relationship between neuronal structure and function across the nervous system. Fluoro-Gold (hidroxistilbamidine) is a fluorescent and retrogradely transported anatomical tracer, which is commonly used to label neurons in the brain and spinal cord for light microscopic fluorescence microscopy [27] and was used to evaluate neuroregeneration. A 0.3 µL injection of Fluoro-Gold was injected with a rate of 0.1 uL/min using a glass micropipette at the level of T13 two weeks before the sacrifice (8 weeks post-injury). The injection on the ventral horns (coordinates: X = ±0.4; Y = T13; Z = −2.5) was performed bilaterally and guided by a stereotaxic apparatus (Stoelting 51730D, Kiel, WI, USA). Neuronal cellular bodies were quantified by calculating the ratio of positive neurons, labeled rostral and caudal, to the injury site (approximately 1 cm from epicenter of the lesion).

### 2.8. Serum Cytokine and VEGF Mediator Analysis

Blood was collected from the tail vein 1, 4 and 8 weeks post-injury and allowed to clot for 30 min before centrifugation (10 min at 10,000× *g*). Then, serum was collected and frozen at −80 °C. An enzyme-linked immunosorbent assay for IL-4, IL-5, IL-13, IL-10, IL-1a, IL-1b, IL-2, IL-6, IL-12p, IL-17, IL-18, IFNg, TNFα and VEGF detection (Millipore, Burlington, MA, USA) was used, and the assay was performed as previously described [28]. Samples were analyzed using a MAGPIX Luminex xMAP^®^ (Austin, TX, USA) instrument. Values for IL-4, IL-5, IL-1a, IL-1b, IL-2, IL-6, IL-12p, IL-17, IL-18, IFNg and TNFα were below the limit of detection and, for this reason, were not analyzed in the results section.

We deep anesthetized animals ten weeks after injury with intraperitoneal sodium pentobarbital (200 mg/mL, Eutasil/Ceva Sante Animale) and trans-cardially perfused with cold 0.9% saline, followed by 300 mL of 4% paraformaldehyde (PFA) in 1× phosphate-buffered saline (PBS). After rough dissection of the vertebral column and spinal cord, tissues were fixed in a 4% PFA solution for 24 h (4 °C). A rough dissection of the vertebral column and spinal cord was performed, and tissues were fixed in a solution of 4% PFA for 24 h (4 °C). The spinal cord was then dissected from the vertebral column and immersed in a cryoprotectant solution (30% sucrose) for 48 h at 4 °C. Afterwards, a 2 cm length of spinal cord tissues, centered on the lesion, was submerged in optimal cutting temperature (OCT) embedding medium, frozen on dry ice and stored at −20 °C. For Fluoro-Gold analysis, we also isolated 1 cm of both rostral and caudal segments immediately next to the 2 cm epicenter of spinal cord tissues. Longitudinal sections of 20 µm thickness were obtained using a cryostat (Leica CM1900, LeicaBiosystems, Wetzlar, Germany) and thaw-mounted onto charged microscope slides (Superfrost Plus, Thermo Scientific, Waltham, MA, USA). Fluoro-Gold staining was analyzed using transversal sections. All histological procedures and evaluations were performed in a blind manner to the treatment groups.

### 2.9. Immunohistochemistry Protocol

For immunofluorescence staining, slices were washed with PBS, permeabilized with 0.2% Triton X-100 for 10 min, and blocked with 5% fetal calf serum in 0.2% Triton X-100 for 30 min. Then, the following primary antibodies were incubated overnight at room temperature (RT): rabbit anti-GFAP for astrocytes (1:200; Dako, Singapore), rabbit anti-IBA1 for microglia and macrophages (1:1000; Pharmingen, San Diego, CA, USA), chicken anti-B3 tubulin for Neurons (1:100; Millipore, Burlington, MA, USA), mouse anti-CREB (1:100; Santa Cruz, Santa Cruz, CA, USA) and rabbit anti-cAMPKA (1:1000; Abcam, Cambridge, UK). The following day, primary antibodies were then probed (3 h incubation) with the appropriate Alexa 594- or Alexa 488-conjugated secondary antibodies (1:1000; Invitrogen). Sections were counterstained with DAPI for 30 min (1:1000; Sigma) and mounted with Immu-Mount^®^ (Thermo Scientific). Between steps, five washes with PBS (1×) were performed. For all immunofluorescence procedures, appropriate negative controls were obtained by omitting the relevant primary antibodies. Images were acquired using a confocal point-scanning microscope (Olympus FV1000). All images were analyzed using ImageJ and FIJI software.

### 2.10. Immunofluorescence Analysis

Spinal cord immunostaining was analyzed by collecting photomicrographs of entire longitudinal slices with the cavity in the center. After obtaining micrographs through confocal microscopy, the photos were opened using the Image J software. The immunofluorescence quantification in each photomicrograph was assessed by the positive staining area for IBA1 and the negative staining area (cavity) for GFAP. For IBA1^+^, we measured the percent of reactive microglia (ameboid microglia), which was calculated by dividing the area of ameboid microglia by the total area of the spinal cord slice. For GFAP, the data plotted in the graphs represent the mean area of the cavity per section. For the spared tissue analysis, the preserved tissue area present in the injury epicenter was automatically segmented based on automatic intensity thresholding and normalized to the total epicenter area. The analysis of the GAP-43 regenerating fibers was conducted on whole-slice mosaic images. Rectangular ROIs were drawn every 200 μm from the epicenter in both directions of the rostro–caudal axis. GAP-43 labeling area was quantified after intensity thresholding, and the value was normalized by the area of the spinal cord within each ROI.

### 2.11. Statistical Analysis

Statistical analysis was performed using the GraphPad Prism 8.00 software. The normality of the data was evaluated using Kolmogorov–Smirnov normality test. Data from the BBB test were assessed using two-way ANOVA test. Differences between groups were compared using the post hoc Bonferroni test. Activity box test, Gridwalk test, immunofluorescence and cytokine concentration data were analyzed using Student’s *t*-test or Mann–Whitney according to normality test. Statistical significance was defined as *p* < 0.05 (95% confidence level). Data are shown as mean +/− standard error (SEM).

## 3. Results

### 3.1. Roflumilast Supports Survival and Increases Nuclear CREB Labeling in Spinal Cord Neurons In Vitro

**Figure 2:**
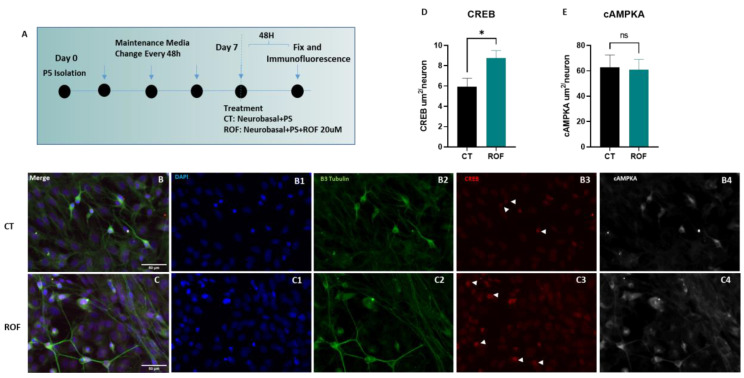
The effect of Roflumilast on cAMP-driven responses in spinal cord neurons. (**A**) Experimental outline. (**B**,**C**) Representative photomicrographs of controls and Roflumilast-treated cells, respectively. (**D**) Neuronal nuclear CREB area quantification. Unpaired *t*-test, 6df, *p* = 0.0441. Controls *n* = 4, Roflumilast *n* = 4. (**E**) Neuronal cytoplasmic cAMPKA area quantification. * *p* < 0.05; ns = non-significant.

To understand if Roflumilast could mediate cAMP-driven plasticity responses, we treated spinal cord neurons derived from Wistar Han pups at (P5) after 7 days in culture (Figure 2A). Using immunofluorescence, we assessed the expression of (cAMPKA) and (CREB) after 48 h of Roflumilast treatment and respective control (Figure 2B,C). The quantification of nuclear CREB and cytoplasmic cAMPKA demonstrated an increase in nuclear CREB translocation marked by an enhanced neuronal nuclear CREB staining area (8.767 ± 0.7375 vs. 5.942 ± 0.8331), while cAMPKA levels were unchanged when compared to controls (Figure 2D,E, *p* = 0.0441).

### 3.2. Roflumilast Administration Promotes Axonal Growth In Vitro

**Figure 3:**
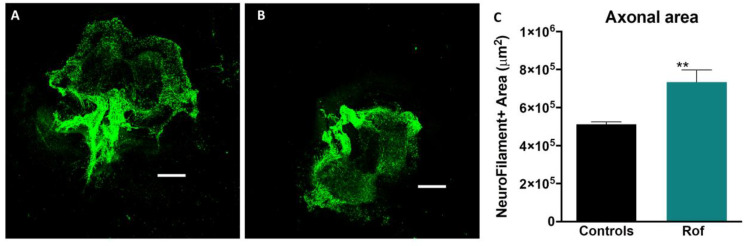
The effect of Roflumilast on axonal regeneration in vitro. (**A**) Neurofilament + staining for cultures with Neurobasal + Roflumilast. (**B**) Neurofilament + staining for cultures with only Neurobasal. (**C**) Neurofilament + area in organotypic cultures. Unpaired *t*-test, 11df, *p* = 0.008. Controls *n* = 4, Roflumilast *n* = 5. ** *p* < 0.01.

We used an organotypic culture of spinal cord slices (350 µm) from the thoracic region of Wistar Han pups within 6 days (P6) to assess the effect of Roflumilast on axonal regeneration. The cultures were incubated with basal medium + 20 nM Roflumilast or basal medium alone. The spinal cord slices cultured with Roflumilast showed higher levels of neurofilament expression (Figure 3A,B), as measured by the total area of positive staining (735,600 ± 12,862 µm^2^), indicating a superior axonal regeneration in this group when compared to controls (512,635 ± 62,363 µm^2^). These results showed that Rof promotes axonal regeneration in spinal cord neurons (Figure 3C; *p* = 0.008).

### 3.3. Roflumilast Treatment Increases Cyclic AMP Concentration In Vivo

**Figure 4:**
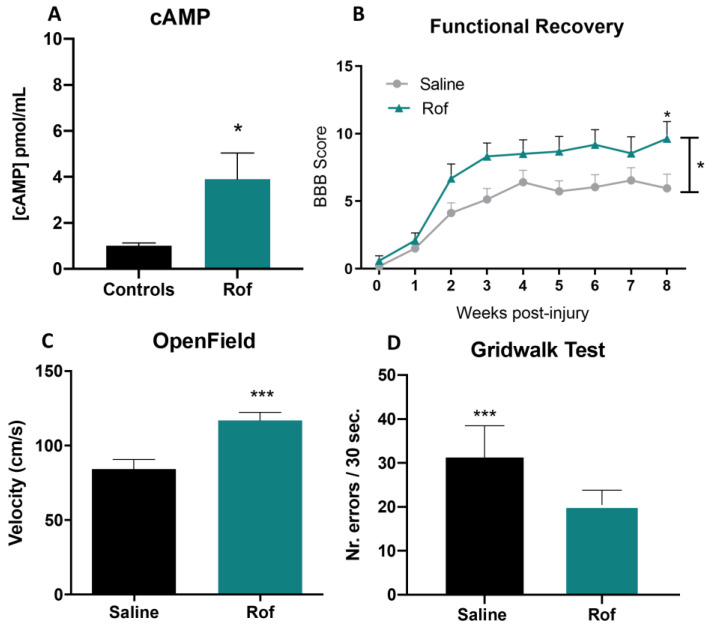
Pre-clinical evaluation of Roflumilast using a contusion SCI model. (**A**) In vivo cAMP concentration for Rof-treated animals and controls. Unpaired *t*-test, 10 df, *p* = 0.029. Saline *n* = 6, Roflumilast *n* = 6. (**B**) Functional recovery after spinal cord injury of Rof-treated animals and controls. Two-way ANOVA F (1, 20) = 4.408, *p* = 0.0487. Saline *n* = 11; Roflumilast *n* = 12. (**C**) Activity box test—8th week. Velocity (cm/s) of Rof-treated animals and controls. Unpaired *t*-test, 21 df, *p* = 0.0008. Saline *n* = 11; Roflumilast *n* = 12. (**D**) Gridwalk test of Roflumilast-treated animals and controls—8th week. Unpaired *t*-test, 21 df, *p* = 0.0001. Saline *n* = 11; Roflumilast *n* = 12. * *p* < 0.05; *** *p* < 0.001.

ELISA analysis, performed on the spinal cords of injured animals, showed a significantly higher concentration of cAMP in rats treated with Roflumilast than in control animals. cAMP concentration in the Rof group was 3.85-fold higher than that in controls (Figure 4A; *p* = 0.029), showing that Rof successfully elevated the levels of cAMP in the spinal cord.

### 3.4. Roflumilast Treatment Promotes Functional Recovery in a Thoracic Contusion Injury Model

Functional recovery was analyzed using the BBB score, ABT and Gridwalk test. The BBB test was performed weekly and revealed that although both Roflumilast (Rof) and saline-treated animals presented a spontaneous recovery over time, Rof-treated animals presented a significantly better functional recovery at 8 weeks post-injury when compared to saline treatment (Figure 4B; *p* = 0.048). At week 8, Rof-treated rats presented a mean BBB score of 9.6 ± 3.7, corresponding to the ability to support their own body weight and to perform occasional plantar steps, while the saline-treated group presented a mean BBB score of 6.0 ± 3.5, which is translated into extensive movement of two joints and a slight movement of the third.

Locomotor velocity was evaluated using ABT 8 weeks post-injury. Rof-treated animals presented better locomotor abilities than saline-treated rats. Rof presented a mean velocity of 116.9 ± 5.35 cm/s, while the saline-treated group presented a mean of 84.2 ± 6.50 cm/s velocity (Figure 4C; *p* = 0.0008).

Finally, the Gridwalk test showed that the number of errors per 30 s of continuous locomotion on top of the wire mesh was superior in saline-treated animals, with a mean score of 31.7 ± 2.10 errors per 30 s, when compared to the Rof-treated group, which showed a mean score of 20.5 ± 0.96 errors per 30 s (Figure 4D; *p* = 0.0001).

### 3.5. Roflumilast Treatment Induces an Anti-Inflammatory Environment

**Figure 5:**
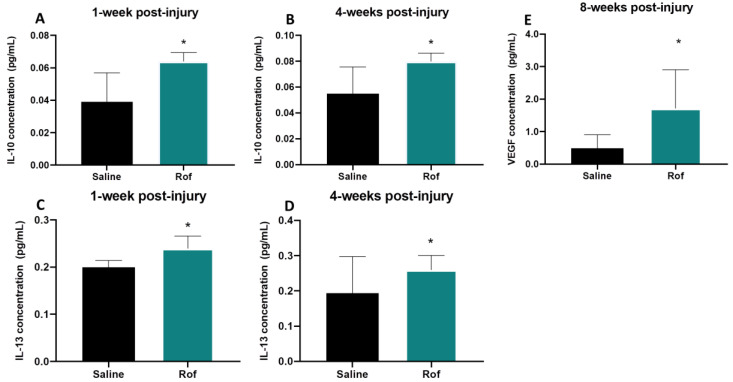
Multiplex analysis. (**A**) Serum IL-10 concentration 1 week post-injury. Unpaired *t*-test, 13 df, *p* = 0.010. Saline *n* = 10; Roflumilast *n* = 5. (**B**) Serum IL-10 concentration 4 weeks post-injury. Unpaired *t*-test, 14 df, *p* = 0.013. Saline *n* = 10; Roflumilast *n* = 6. (**C**) Serum IL-13 concentration 1-week post-injury. Unpaired *t*-test, 6 df, *p* = 0.035. Saline *n* = 4; Roflumilast *n* = 4. (**D**) Serum IL-13 concentration 4 weeks post-injury. Unpaired *t*-test, 7 df, *p* = 0.027. Saline *n* = 5; Roflumilast *n* = 4. (**E**) Serum VEGF concentration 8 weeks post-injury. Unpaired *t*-test, 9 df, *p* = 0.040. Saline *n* = 6; Roflumilast *n* = 5. * *p* < 0.05.

Blood samples were collected and the serums were analyzed 1, 4 and 8 weeks post-injury. Cytokine quantification was performed by multiplex analysis. Results revealed that IL-10 and IL-13 were significantly increased in the serum of Roflumilast-treated animals at the first and fourth weeks post-injury (Figure 5A–D). The serum concentration of IL-10 in Rof-treated rats was 1.6-fold higher than that in saline-treated animals 1 week post-injury (Rof: 0.064 ± 0.002 pg/mL; Saline: 0.039 ± 0.006 pg/mL) and 1.5-fold higher 4 weeks post-injury (Rof: 0.080 ± 0.003 pg/mL; Saline: 0.055 ± 0.007 pg/mL;). The elevation in the serum concentration of IL-13 was also 1.2-fold (Rof: 0.240 ± 0.013 pg/mL; Saline: 0.200 ± 0.007 pg/mL;) and 1.3-fold (Rof: 0.260 ± 0.020 pg/mL; Saline: 0.194 ± 0.046 pg/mL) higher 1 week and 4 weeks post-injury, respectively, in the Rof group. Moreover, the results also demonstrated that VEGF was significantly higher (3.5-folds) at 8 weeks post-injury in the Rof-treated group (1.720 ± 0.530 pg/mL) versus the controls (0.490 ± 0.170 pg/mL) (Figure 5E; *p* = 0.040).

### 3.6. Roflumilast Treatment Reduces Cavity Size, Increases the Spared Tissue and Reduces Microglia Reactivity

**Figure 6:**
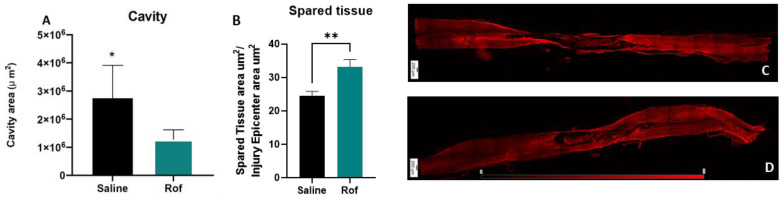
(**A**) Cavity Size of Roflumilast-treated animals and controls. Unpaired *t*-test, 12 df, *p* = 0.0149. Saline *n* = 8; Roflumilast *n* = 6. (**B**) Spared tissue in the injury epicenter of Roflumilast-treated animals and controls. Unpaired *t*-test, 12 df, *p* = 0.0036. Saline *n* = 8; Roflumilast *n* = 6. (**C**) Longitudinal GFAP+ staining slice of saline-treated group and (**D**) for Rof-treated group. A total of 69 longitudinal slices were analyzed. * *p* < 0.05; ** *p* < 0.01.

**Figure 7:**
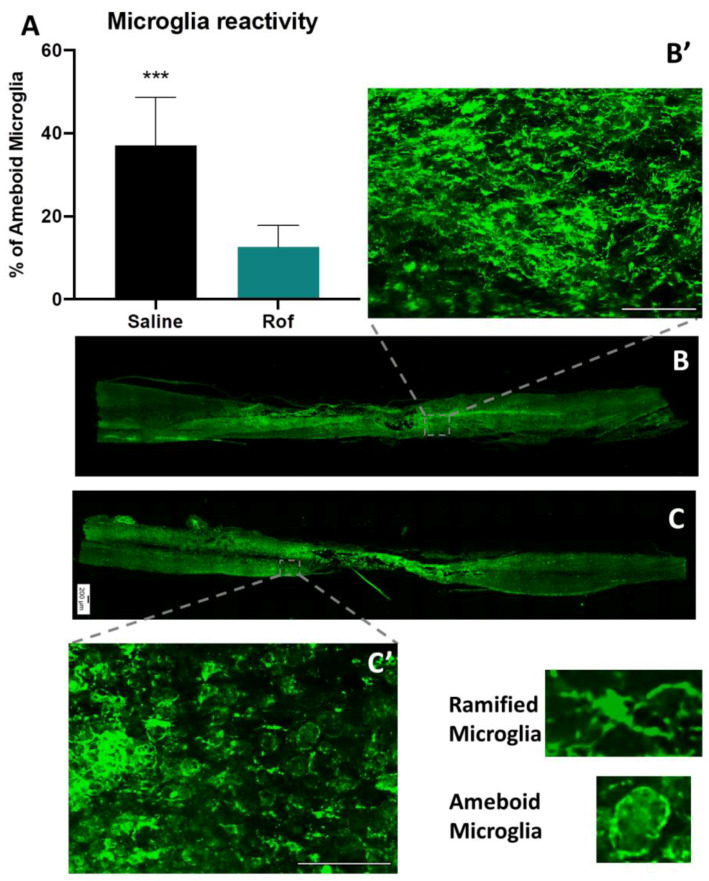
(**A**) Microglia staining with IBA1 + to assess the percentage of ameboid microglia in Roflumilast-treated animals and controls. Unpaired *t*-test, 12 df, *p* = 0.0003. Saline *n* = 7; Roflumilast *n* = 7. (**B**) Longitudinal slice of injured spinal cord, with IBA1+ staining for microglia reactivity in a Rof-treated animal; (**B’**) magnification of the dashed square. (**C**) IBA1 + staining in an injured spinal cord of a saline-treated animal; (**C’**) magnification of the dashed square. A total of 68 longitudinal slices were analyzed. *** *p* < 0.001.

Histological analysis was performed 10 weeks post-injury. Lesion size and spared tissue were quantified using GFAP^+^ staining (Figure 6C,D). An analysis of longitudinal slices of spinal cord tissue revealed that Rof treatment promoted a global reduction of lesion size when compared to the saline-treated group (Rof: 1,286,633 ± 138,341 µm^2^; Saline: 2,745,543 ± 413,518 µm^2^. Figure 6A; *p* = 0.0149) and led to significant increases in the spare tissue (Rof: 33.23 ± 2.14 µm^2^; Saline: 24.50 ± 1.36 µm^2^. Figure 6B; *p* = 0.0036). In relation to microglia reactivity, we found that the percentage of IBA1 positive cells (Figure 7B,C) with amoeboid morphology was significantly lower (12.55 ± 1.97%) in the Rof-treated animals when compared to the controls (37.03 ± 4.41%) (Figure 6A; *p* = 0.0003).

### 3.7. Roflumilast Treatment Increases Neuronal Regeneration

**Figure 8:**
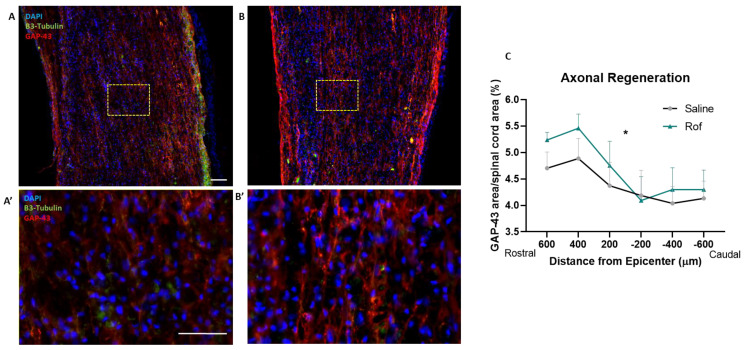
Roflumilast increases GAP-43 expression along the rostro–caudal axis of the injured spinal cord. (**A**,**B**) Microphotographs of the spinal cord of control and Roflumilast-treated animals. (**C**) Shows the quantification of GAP-43 fibers along the rostro–caudal axis at 200, 400 and 600 μm from the injury epicenter in both directions. Two-way ANOVA F (DFn:1.409, DFd:18.31) = 4.182. Saline *n* = 7; Roflumilast *n* = 8. Insets show zoom magnification GAP-43 labeling. Scale Bars: 250 μm at (**A**,**B**) and 50 μm at (**A**’,**B**’). * *p* < 0.05.

In order to analyze axonal regeneration in the spinal cord tissue, we performed immunohistochemistry against Growth-Associated Protein 43 (GAP-43). Our analysis demonstrated that animals treated with Roflumilast have a statistically higher expression of GAP-43, namely on the rostral side of the spinal cord of injured animals (Figure 8; *p* = 0.0437).

**Figure 9:**
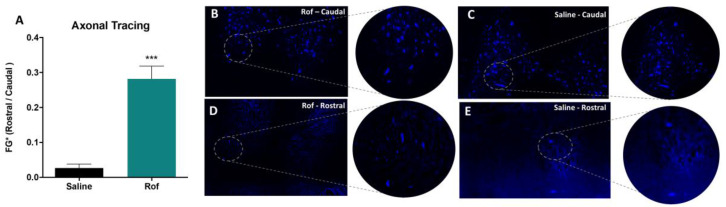
Roflumilast promotes axonal regeneration in vivo. (**A**) Fluoro-Gold ratio (Rostral/Caudal) in Roflumilast-treated animals and controls. Unpaired *t*-test, 12 df, *p* < 0.0001. Saline *n* = 7; Roflumilast *n* = 7. (**B**) Fluoro-Gold + staining in a Roflumilast-treated animal caudal to the epicenter. (**C**) Fluoro-Gold + staining caudal to the epicenter in a saline-treated animal. (**D**) Fluoro-Gold + staining in the Roflumilast group rostral to the lesion. (**E**) Fluoro-Gold + staining rostral to the lesion in the saline group. A total of 88 transversal slices were analyzed. *** *p* < 0.001.

Fluoro-Gold, a fluorescent retrograde tracer, was also used to evaluate neuroregeneration. The tracer was stereotaxically injected (bilaterally) on the ventral horns, at T13, eight weeks post-injury. Staining was analyzed in cross-sections of the spinal cord, rostral and caudal, to the injury site (approximately 1 cm from the lesion epicenter). Neuronal cellular bodies were quantified by calculating the ratio of positive neurons, labeled rostral and caudal, to the injury site (FG^+^ rostral/FG^+^ caudal). The Fluoro-Gold analysis revealed higher positive staining on Rof-treated animals (0.280 ± 0.036) at the rostral extremity of the thoracic spinal cord when compared to the control group (0.027 ± 0.011), supporting the neuroregenerative effect of Rof treatment (Figure 9; *p* < 0.0001).

## 4. Discussion

Despite great advances in knowledge about the processes that regulate axonal regeneration, the failure of axons to properly regenerate after spinal cord injury remains a critical challenge. One of the most important breakthroughs understanding axonal regeneration has been the identification of inhibitory proteins in CNS myelin (such as MAG, Nogo and OMgp) [11,26,27,28,29] and the development of strategies that enable axons to overcome myelin inhibition. It has been demonstrated that there is a correlation between neuronal cAMP levels and the inhibition of neurite outgrowth by MAG and myelin. Therefore, when levels of endogenous cAMP are high, substantial neurite outgrowth can be observed, even in the presence of MAG and myelin. However, in case of a decrease, there is the onset of myelin inhibition [10]. These findings suggest that pharmacological agents can be used to elevate cAMP levels in neurons and increase their ability to regenerate.

Inhibiting the PDE4 receptor, which is responsible for cAMP hydrolysis, is one of the targets for increasing cAMP levels. PDE4 inhibition increases the levels of cAMP, which activates protein kinase A (PKA) and successively CREB, thereby promoting the transcription of genes associated with synaptic plasticity, axonal growth and neurogenesis, such as the neurotrophic factor BDNF [15] and Arginase 1 [30]. The beneficial potential of PDE4 inhibition was first tested using Rolipram. The administration of this drug to SCI rats significantly improved axonal integrity and functional outcomes [12,13,14]. These results demonstrated the therapeutic potential of PDE4 inhibition. However, Rolipram, which was originally developed as an antidepressant drug in the early 1990s, has since been discontinued due to a narrow therapeutic window with significant gastrointestinal side effects as well as nausea and emesis [15,31]. In this sense, it is critical to find alternatives to Rolipram that can be safely tested in persons with SCI. The purpose of this study was to propose Roflumilast as a neuroregenerative drug for treating SCI. Previous studies have demonstrated that Rof, like Rolipram, is also a PDE4 inhibitor that leads to an increase in cAMP [32]. Additionally, Rof presents excellent pharmacokinetic properties, such as rapid absorption and distribution, linear kinetics, significant bioavailability and low toxicity [16], and is approved for human use in COPD [17]. Indeed, Roflumilast is currently the only PDE4 inhibitor approved for the treatment of severe COPD in humans [32]. Taking this into account, we tested the therapeutic potential of Rof in repairing the injured spinal cord. First, to understand whether Rof can promote axonal regeneration, we used organotypic cultures of the spinal cord from the thoracic region. The in vitro results demonstrated that Rof treatment promotes axonal regeneration in spinal cord slices, showing that Roflumilast creates a favorable neuroregenerative environment. We also analyzed axonal regeneration in the spinal cord tissue by performing immunohistochemistry against Growth-Associated Protein 43 (GAP-43), and the animals treated with Roflumilast have a statistically higher expression of GAP-43 in the rostral side of the spinal cord. GAP-43 is enriched in growth cones and presents itself at high levels during axonal elongation [29], playing a critical role in axonal regeneration [30,31].

As we know, the transected axon is not inherently immobile but is in a latent state of growth, which is suppressed by factors of the local environment. As a result of blocking cAMP hydrolysis, Roflumilast attenuates the effects of inhibitory factors and restores the status of permanent growth inhibitory status. This effect was described for Rolipram [33]. Moreover, elevating neuronal cAMP levels, using db-cAMP or through a conditioning lesion to DRG neurons, has also been demonstrated to promote axonal growth [34,35]. Our results are another example of this mechanism being essential for axonal regeneration. However, we demonstrated this by using a drug that can be safely administered in humans, avoiding the side effects of Rolipram or db-cAMP.

To understand whether Rof administration was able to elevate the levels of cAMP in the spinal cord of living animals, we performed molecular analyses and demonstrated that Rof increased the tissue concentration of cAMP in the injured spinal cord when compared to saline-treated animals. The elevation of cAMP caused by Roflumilast can be considered a bypass to the neurotrophin-activated signaling cascade [36] with the consequent activation of pathways such as cAMP/PKA/CREB [36,37,38,39,40] and cAMP/Epac 2 [39], which ultimately leads to axonal regeneration [36,37,38,39]. CREB-dependent transcription has been shown to control axon regeneration in both the PNS and CNS [32,34]. On the other hand, the expression of a dominant negative form of CREB has been shown to block axonal growth [36]. Herein, we assessed the pathway cAMP/PKA/CREB by evaluating the expression of (cAMPKA) and (CREB) after 48 h of Roflumilast treatment. We demonstrated an increase in nuclear CREB translocation marked by an enhanced neuronal nuclear CREB staining area in spinal cord neurons, while cAMPKA levels were unchanged when compared to controls. We then tested if the administration of Rof leads to an increase of cAMP in the spinal cord tissue. The increase in cAMP levels in Rof-treated animals was expected considering the mechanism of action of this drug [38,39,41,42,43,44]; however, it worked as a confirmation step before testing Rof administration in large animal sets.

To test the therapeutic effect, using a contusion injury model at the thoracic level (T8), we observed that Rof treatment promoted locomotor recovery. When provided with Rof, SCI rats were able to achieve weight support and perform occasional plantar steps. In contrast, control animals were only able to perform extensive movements of two joints and a slight movement of a third joint. This improvement in hindlimb motor skills is probably the reason why the treated animals also walked over the ground faster than the non-treated animals. These results suggest that Rof treatment has a beneficial effect on incomplete paraplegia.

To explore the impact of Rof’s therapeutic effect on the restoration of fine motor skills, we also performed the Gridwalk test. During this test, the animals were required to place their limbs on a grid wire and walk. Effective achievement of this task demands the activation of more than one neuronal pathway. It involves the coordination of the forelimb and hindlimb, which is mediated by ventrolateral tracts [45,46]; recruits the stepping rhythm, which implies a functioning reticulospinal system; and involves voluntary movement control, which is mainly mediated by the corticospinal tract [47,48]. The results demonstrated that Rof-treated animals recovered fine motor skills to a greater extent than saline-treated animals.

Subsequently, we analyzed the long-term effects of Rof treatment at the histological level. Histological analysis of thoracic-injured animals demonstrated that Rof treatment considerably reduced the cavity size and also increased the spare tissue volume, suggesting a sustained protective effect on spinal cord tissue.

Moreover, histological analysis revealed that the inflammatory response in spinal cord tissue was significantly reduced with Rof treatment. The persistent and non-resolving immune response after SCI is an important contributor to the secondary damage observed after primary injury. At the initial stages of SCI, the immune response resembles that in non-CNS injured tissue [33]. However, pro-repair immune cells are unable to populate the wounded tissue, and pro-inflammatory cells quickly become the predominant type at the injury site [35]. This immune response is associated with the expression of pro-inflammatory cytokines, fibrosis, oxidative damage and neurodegeneration, contributing to wound-healing failure [37]. The reduction in ameboid microglia/macrophages is probably due to a direct effect on inflammation by Rof since it has previously been demonstrated that Rof has an anti-inflammatory effect [48,49]. For instance, as seen in the lung, Rof consistently minimizes the release of TNF-α, CCL2, CCL3, CCL4, CCL5 and CXCL9 from human bronchial explants [49]. The accumulation of microglia/macrophages in the lesion epicenter plays a significant role in the inflammatory response during secondary injury. Microglia/macrophages are divided into two main subtypes: the pro-inflammatory subtype, which promotes inflammation and may lead to further tissue damage, and the pro-regenerative subtype, which promotes anti-inflammatory reactions, resolution of inflammation, and tissue repair [50,51]. Elevated intracellular cAMP levels in macrophages exhibit immunosuppressive activity predominantly by antagonizing proinflammatory cytokine production and phagocytosis [52]. Moreover, a previous study has shown that Roflumilast not only significantly decreases the M1/M2 ratio but also reduces the levels of pro-inflammatory molecules in an injured spinal cord [53]. These results led to the hypothesis that cAMP elevation may reduce secondary injury caused by local inflammation. Herein, we also examined the serum cytokines of spinal cord-injured rats, and the analysis revealed that IL-10 and IL-13 levels were increased in the serum of Roflumilast-treated animals at 1 and 4 weeks post-injury. IL-10 is an anti-inflammatory cytokine, and several studies performed over the past few decades have revealed positive effects of IL-10 treatment post-SCI [54]. IL-10 treatment has been shown to protect against secondary inflammation by downregulating pro-inflammatory cytokines [55,56,57] and provides trophic support and neuroprotection [58], leading to functional recovery in rat models [59].

IL-13 is also an anti-inflammatory cytokine that has been reported to protect against demyelination and spinal cord injury through immunomodulation. IL-13 suppresses pro-inflammatory responses and enhances the phagocytic capacity of microglia/macrophages [60]. It was also seen that IL-13 shared many functions with IL-4. Interleukin-4 and IL-13 are signature cytokines of a type II inflammatory response. They are key players in the inflammatory response triggered by either an invading parasite or an allergen. Cellular sources of IL-4 and IL-13 have been studied extensively, and, along with CD4+ T cells, basophils, eosinophils, mast cells and NK+ T cells, appropriately stimulated ILC2 cells have the ability to produce IL-4 and IL-13 [61,62,63]. Recently, IL-4 has emerged as a promising therapeutic strategy in the context of SCI due to its ability to promote the M2-alternative activation of macrophages [64,65], a phenotype associated with neuroprotection and repair [19,66]. Additionally, our team also showed that IL-4 treatment increased circulating levels of the anti-inflammatory cytokine IL-10 and reduced inflammatory markers in the spinal cord of injured rats [18].

We also observed that VEGF levels were 3.5-fold higher at 8 weeks post-injury in the Roflumilast-treated group (*p* = 0.040). VEGF is a mitogen-activated protein that plays an active role in the formation of new blood vessels and cellular processes, such as proliferation, growth and survival. Importantly, VEGF’s role in the nervous system may be related to neurogenesis [67], neuromaturation [68], neuroprotection [68,69,70,71] and neuroregeneration [72]. This effect occurs through the VEGF receptor and MAPK/Erk1/2 signal transduction pathways, which mediate growth effects on CNS neurons [69]. As such, VEGF promotes the reorganization of the spinal motor network after SCI [73] and has a protective effect against ischemia [74]. On the other hand, it is important to note that there is a possible link between VEGF to nonspecific axonal sprouting in dorsal horns, dorsal columns and mechanical allodynia after SCI [75]. Some studies have also shown that treatment with angiogenesis inhibitors suppresses peripheral neuropathy-induced central angiogenesis, neuroinflammation, astrocyte activation and neuropathic pain [75]. Therefore, the role of VEGF must be further evaluated to balance the benefits and constraints.

To understand if our treatment promotes axonal regeneration through the lesion site, we used the tracer Fluoro-Gold, which has been demonstrated to undergo retrograde axonal transport. We injected the tracer caudally into the injury site and quantified neurons, labeled rostral, to the lesion. As a result, the tracer needed uptake and traveled through regenerated fibers at the injury site. Using the contusion injury allows us to test our therapy in a relevant model; however, it is not the ideal model to test regenerating axons. Full transections models are more appropriate to test axonal regeneration because they fully ablate the ascending and descending information; however, this model is not clinically relevant. In order to have confidence in FG results, it is important that all animals were equally injured, and our locomotor analysis 3 days post-injury shows that all animals presented total paralysis of the hind limbs. In this work, we also normalized the number of labeled neurons for each animal. By performing the analysis and calculating the ratio of caudal-labeled neurons with the rostral-labeled neurons, we nullify the interference of the injection in each animal. All animals were injected in the same place of the spinal cord. However, microscopic variabilities in the injection site may have an impact on the amount of tracer uptake and retrograde transportation. Calculating the rostral/caudal FG-labeled neurons ratio shows us that treatment promoted higher axonal regeneration or higher neuronal plasticity. It is important to point out that the same retrograded tracer was used in previous incomplete SCI models [76,77,78,79]. Considering this, we showed that Rof-treated animals had higher positive staining in the rostral sections of the thoracic spinal cord compared to the control group. These results support the neuroregenerative effect of Roflumilast.

## 5. Conclusions

In this study, we demonstrated that Rof is a promising therapeutic drug for acute SCI management. Our results demonstrated the beneficial effects of Rof treatment on both functional recovery and neural regeneration. Considering the therapeutic effect of Rof, the clinical relevance of our findings, the translational potential of this therapeutic strategy and Rof’s track record for safety, we believe that Rof has the potential to be applied in SCI clinical practice. 

## Data Availability

The data that support the findings of this study are available from the corresponding author upon reasonable request.
